# Supplementary material for: Modeling the interplay of sex hormones in cardiac hypertrophic signaling
Source: PLoS Comput Biol. 2025 Dec 10;21(12):e1012895. doi: 10.1371/journal.pcbi.1012895 (PMC12704863; doi:10.1371/journal.pcbi.1012895)
Supplement: Attachment — Submitted filename: Review.pdf [file pcbi.1012895.s002.pdf]

This manuscript presents an intracellular signaling network model that include the effects of sex hormones to investigate sex-differences in cardiac hypertrophy. The strengths of this work are the novelty of considering sex hormones and the attempt to quantitatively validate the model. However, I found the manuscript difficult to follow. I believe most of this could be fixed by including more details in the text, especially given its structure in including the results before the methods. Further, paper is quite descriptive and further interpretation of what the results mean (particularly biological) would strengthen the paper. Below are my specific comments/questions:

1. In the text, the mechanical stimulus is referred to as "Strain", but the model schematic refers to "Stretch". Please fix.

Qualitative validation:

2. For this section and the "Quantitative Validation" below, did you look at the steady-state response of the network model? If so, please indicate in the text.

3. Page 4, Lines 112-113: "The quality of fit of the response was determined by comparing the decrease ($\Delta y<-0.05$), increase ($\Delta y>0.05$), or no change ($-0.05 \leq \Delta y \leq -0.05$) of downstream node activity with literature data." Please specify the predicted increase/decrease from which simulation. More details on how you compared this quantitative measure to the qualitative literature data would be helpful. Were these the threshold to determine statistical significance? Please explain.
4. Ryall, et al [17 in the manuscript] has published a comprehensive model of cardiomyocyte hypertrophy that includes the same pathways as in your model. Does your model behave similarly to the Ryall model, for example if you simulate an increase in the AngII, ET-1, or Strain?

5. I found it difficult to follow your results along with Figure 2. I would recommend labeling each group of simulations as panels.

6. While the Figure 2 caption explains how the relative changes were calculated (i.e., relative to baseline or relative to a single stimulus), this explanation was missing in the text (Qualitative validation) Please include. (See also Comment 1 above)

7. In your qualitative validation, were there any disagreements that stood out? Were these disagreements due to a completely opposite prediction (for example, predicted up-regulation but reported down-regulation), or because the model under or over-predicted a species activity? I would also recommend labeling the boxes in Figure 2 that disagreed with the literature data, especially since there is only 4 of them.

Quantitative Validation:

8. How did you determine how much to increase each input for these studies? Some details are included in the methods, but the choice seems qualitative without

considering how they quantitatively impact CellArea. Is it possible to calibrate the model inputs to quantitatively match any of the data used to build the model?

9. Were the different concentrations of hormones (for example, E2) in the different studies accounted for?
10. AngII is known to increase in response to aortic banding. Was this increase accounted for in the Donaldson study?
11. Similarly, was strain increased in the spontaneously hypertensive rat study? This was not clear from the text. Were these studies in male or female rats? Also, which bars in Fig.3b refer to the Donaldson vs. Chen data?
12. One concern is that we wouldn't expect a significant change in "Strain" in response to aortic banding, but instead an increase in stress. I would be careful with the wording to emphasize that this is a generic mechanical input (earlier on in the manuscript)
13. Were the in vivo studies in ovariectomized or intact females? Were the expected concentrations of E2 accounted for to reflect this?

Cross-talk between hypertrophic stimuli

14. Did you validate or is it possible to validate some of these cross-talk predictions?

Case Studies: comparisons between sex and age

15. I had a hard time understanding the Influence and Sensitivity analysis in Figure 5, as well as the description in the text. I would recommend re-organizing this section. For example, focusing on the influence first, then sensitivity after, etc.
16. What does it mean biologically that T is most influential? I understand that computationally, up-regulating T will influence the activity of all other species the most. Does this mean that you would expect Testosterone to have a largest effect on cardiac hypertrophy compared to other inputs/species? Please explain the significance of this analysis.
17. "T's higher influence when compared to E2 suggested by this model is important in explaining the differential response to cardiac hypertrophy in pre-menopausal females, post-menopausal females and males, as well individual differences within these categories." What does this mean? What data do you have to support that T has a higher influence compared to E2 clinically?

Discussion:

18. An important implication of your model could be explaining why hormone replacement therapies are controversial for cardiac health in post-menopausal women (https://www.physiology.org/doi/10.1152/ajpheart.00041.2018) or possibly for gender affirming care. Are there any insight gained from your model for informing/explaining the effects of hormone replacement therapies?
