# Supplementary material for: Modeling the interplay of sex hormones in cardiac hypertrophic signaling
Source: PLoS Comput Biol. 2025 Dec 10;21(12):e1012895. doi: 10.1371/journal.pcbi.1012895 (PMC12704863; doi:10.1371/journal.pcbi.1012895)
Supplement: Attachment — Submitted filename: Review_ Modeling the Interplay.pdf [file pcbi.1012895.s003.pdf]

- In lines 86-87 (“We opted for a single composite output, CellArea…”) while this makes sense in order to have a directly quantifiable output for what you wish to measure - increase or decrease of cell size - it does obscure a bit the network and how the ODEs apply to it. This is because now the nodes do not represent only biomolecules (line 73). The same occurs with having Strain as an input node. In particular, it is not entirely clear to me what a CellArea = 0 means (or Strain = 1). Neither is the physical mechanism for the nodes to “interact” with CellArea, i.e. Hill Functions are usually explained in terms of the concentration levels of the biomolecules with the function parameters representing the different physical interactions between them (e.g. binding). This needs to be directly addressed in the main text.
- In lines 80-81, as well as 316-317, it is explained that because E2 and T “are linked to a similar number of downstream nodes” there is no connectivity bias as to their downstream influence. However the degree of a node alone (or even the *number* of nodes in its downstream paths - which is what you seem to be referring to) does not determine the influence or centrality of a node. Two nodes A and B can be connected to the same amount of nodes but if node A's neighbors lead to nowhere whereas B’s neighbors do, then it follows that node B will have a bigger influence in network dynamics. The focus should be the different pathways from the input nodes to the output node CellArea. I recommend to include a simple analysis of node centrality or influential nodes in the network. This can also be a useful comparison to results in Fig 5.
  By looking at Fig 1 it seems that for the most part T and E2 downstream paths rarely cross, except at nodes eNOS and ERK12. eNOS seems to act as a “bottle-neck” for multiple pathways from E2 and one pathway from T, which probably explains why it is such an influential node under knock-down analysis (lines 176-178).
- Line 75, as well as 395, mention the use of OR gates but Fig 1 only shows AND gates. Additionally, how do you account for more than 2 inputs received? As in node MEF2 receiving 3 inputs, or even CellArea? Please address this explicitly
- Fig 1 suggestions: I think the figure would be improved if you make visually clear the top-down structure of the network, by putting all input nodes atop, with the intermediate nodes strictly beneath them. Additionally, you can just use a smaller, differently shaped node to stand for AND gates and add it to the legend. This way you avoid the need to put the word AND so many repeated times, removing some visual clutter. Preferably I would also use a different shape for CellArea since it is not an actual biomolecule as the rest of the nodes but more of a standing for the network’s output.
- In lines 96-98 the conflicting behaviour of this specific path by sex is mentioned, could you give a plausible explanation as to how this is the case? Perhaps there

is an intermediate node modulating this different behaviour or something similarly explainable?

- In section **Quantitative validation** (line 125), you write ‘under-predicted’, ‘overestimated’, etc.., but since this is easy to see from Fig 3, perhaps it would be helpful if in the text you write by how much you under/over-estimated instead, which is harder to see precisely from the fig.
- Lines 173-174 mention that CellArea is expected to be the most sensitive node since it is the most downstream node. But this implies that the network is not robust at all with respect to changes in the input/intermediate nodes. Is this biologically desirable? Shouldn’t it be more advantageous for the network to be robust to changes so that perturbations don’t result in hypertrophy?
- Line 205, “exceeds” but barely so, from figure 6 it looks almost the same value.
- Line 279 “we can attribute to increased E2” sounds off, perhaps add levels: “we can attribute to increased levels of E2”.
- Lines 334-337 and 353 talk about the need and impact of including organ-scale models. Could you briefly elaborate how you could extend your model to include these?
- Equations 9 and 10 in subsection **Influence and sensitivity analysis** correspond to two different summations. The variable delta y should actually include two indices: one for the node (the only one explicitly shown) and another one for the case (i.e. different knockdown/upregulation instances). This way the influence of a node is a summation over all nodes (for a given case), while the sensitivity is the summation over all the cases (for that given node).
  I.e. (9) = Sum over i for all y_ij while (10) = Sum over j for all y_ij; i stands for the node and j for the knockdown/upregulation case.
