# Supplementary material for: Modeling the interplay of sex hormones in cardiac hypertrophic signaling
Source: PLoS Comput Biol. 2025 Dec 10;21(12):e1012895. doi: 10.1371/journal.pcbi.1012895 (PMC12704863; doi:10.1371/journal.pcbi.1012895)
Supplement: Attachment — Submitted filename: AKL_PJO_Paper1_rebuttal_2025.pdf [file pcbi.1012895.s004.pdf]

Response To Reviewers

PCOMPBIOL-D-25-00341
Modeling the Interplay of Sex Hormones in Cardiac Hypertrophic Signaling
PLOS Computational Biology

We would like to thank all reviewers for their rigorous and meaningful feedback, which we believe has strengthened our manuscript.

Please note: Since the reviewer numbers were not provided, we have responded to the comments as 'Reviewer 1', 'Reviewer 2', etc., corresponding to the order in which the files were received. These may not correspond to the original reviewer numbers.

Reviewer 1
1. Consider briefly elaborating on the advantages of using the normalized Hill equation — such as its ability to represent saturation effects and cooperative binding — especially in the absence of precise kinetic data.

- We thank the reviewer for evaluating our manuscript and providing constructive comments. We have added a line in the introduction to highlight this advantage lines 39-41:

> "The relationships are modeled by normalized Hill-type ordinary differential equations to capture cooperative binding mechanisms and saturation effects even in the absence of actual kinetic data (Frank et al. 2018, Goutelle et al. 2008)

2. Parameter Sensitivity using Latin Hypercube Sampling (LHS) To further strengthen the parameter sensitivity analysis, you might consider incorporating Latin Hypercube Sampling (LHS). This approach is well-suited for exploring high-dimensional parameter spaces and could offer additional insight into how variations in the assumed Hill-type parameters influence model outputs, particularly given the lack of quantitative data for individual reactions.

- We agree with the reviewer that additional sensitivity analysis is insightful so we added a global parameter sensitivity analysis using Sobol's method, as suggested by Reviewer 4 comment #1. This addition is present as Figure 5(a,b) in the new manuscript along with the local influence and sensitivity analysis Figure 6 (a,b). Note that Sobol's method, like most global sensitivity analyses, assumes that inputs are independent. Because several downstream nodes are dependent on each other through interconnected signaling pathways, we only included the most upstream nodes as inputs in the Sobol analysis.
- The results showed that T and E2 have a higher influence on many downstream nodes when compared to other inputs, with T being more influential on CellArea than E2. We can also see which specific pathways are influenced to a greater extent by Strain, AngII and ET-1, which will be important when understanding the influence of drugs on specific

pathways in the future. This has now been added to the results section under Cross-talk Lines 176-180 of the revised manuscript as :

> "Conducting a global sensitivity analysis using Sobol's method, we observe T and E2 to have a higher influence on many downstream nodes when compared to other inputs, with T being more influential on CellArea than E2. We also see which nodes are influenced to a greater extent by each of the inputs,, which will be important when understanding the influence of drugs on specific pathways in the future."

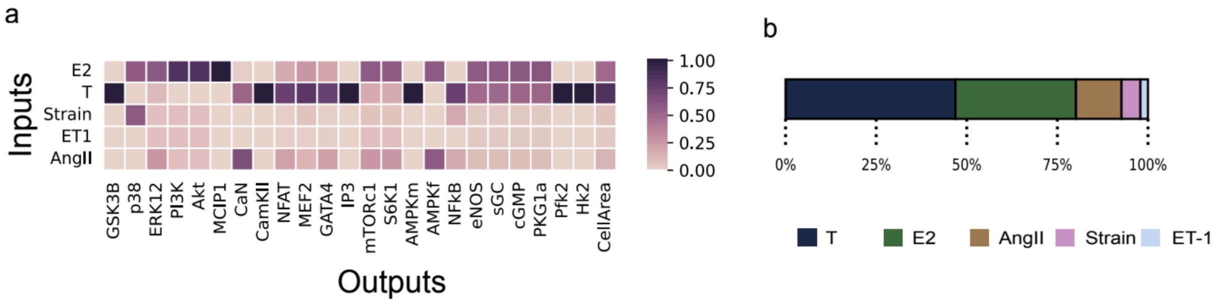

3. Physiological Implications - - References: In both the up-regulation and knockdown analysis paragraphs, please add more citations from previously published literature on the role of CaMKII, eNOS, and GSK3β in cardiomyocytes.

- Thank you for highlighting missing references here. CamKII leads to an increase in calcium signaling in cardiomyocytes, and now includes new references (Anderson et al 2011, and Zhang and Brown 2004). eNOS regulates heart rate, vasodilation and hypertrophy, and now references (Massion and Balligand 2003, Ahmad et al. 2016). GSK3B's role in sarcomere activation also has references included (Stachowski-Doll et al. 2022).The following sentences highlight the changes above in the Discussion Physiological Implications Section, Lines 301-302 and 310-312 of the revised manuscript:

  > "The increased CamKII influence across all models indicates an increase in calcium signaling activity (Anderson et al 2011, Zhang and Brown 2004)", and, "eNOS is a vasodilator that regulates heart rate (Massion and Balligand 2003), and ameliorates progression of left ventricular hypertrophy (Ahmad et al. 2016). GSK3B localizes to the cardiac Z-disc to maintain length-dependent activation in cardiomyocytes (Stachowski-Doll et al. 2022).

4. Possible Future Direction - - Personalization of Input Nodes: The activation or deactivation of T, AngII, E2, and ET-1 nodes may vary based on sex-specific transcriptomic signatures observed at the single-cell level. These signatures reflect gender-differentiated gene expression patterns, which can influence the sensitivity and response of cells to these hormonal stimuli,

ultimately leading to sex-dependent modulation of cellular behaviors such as hypertrophy. Incorporating these variations into the model could provide a more accurate representation of how sex influences the signaling dynamics in cardiac cells. This integration could also help identify novel sex-specific therapeutic targets, ultimately improving the precision of future treatments for cardiac hypertrophy.

- We agree with the reviewer that this is a promising future direction. This has now been added to future directions in the discussion section lines 415-419:

    > “Integrating transcriptomic information into our mode, such as by adjusting reaction weights, in the future could further improve the precision of future treatments for cardiac hypertrophy. Sex-specific transcriptomic signatures observed at the single-cell level lead to differential gene expression patterns, thereby influencing cellular hypertrophic response to the hormonal inputs present in this network model.”

Reviewer 2

1. In the text, the mechanical stimulus is referred to as “Strain”, but the model schematic refers to “Stretch”

- We thank the reviewer for evaluating our manuscript and providing constructive comments. We have addressed this inconsistency by replacing “Stretch” with “Strain” in Figure 1 to be consistent with the text.

2. Regarding Qualitative validation figure and text: For this section and the “Quantitative Validation” below, did you look at the steady-state response of the network model? If so, please indicate in the text. While the Figure 2 caption explains how the relative changes were calculated (i.e.,relative to baseline or relative to a single stimulus), this explanation was missing in the text (Qualitative validation) Please include. I would recommend labeling each group of simulations as panels.

- Thank you for this feedback. We indeed checked if a steady-state was established in this baseline simulation as well in the single perturbation simulations before applying the second perturbation. We now elaborate on this in the text of the Results section on Qualitative validation (Lines 137-139) as:

    > “We ensured that a steady-state was established in the baseline simulation and in the single perturbation simulation before applying further perturbations.”

- We also clarify the addition of the white space as panels in the Fig 2 description.

3. Page 4, Lines 112-113: “The quality of fit of the response was determined by comparing the decrease ($\Delta y<-0.05$), increase ($\Delta y>0.05$), or no change ($-0.05 \leq \Delta y \leq -0.05$) of downstream node

activity with literature data." Please specify the predicted increase/decrease from which simulation. More details on how you compared this quantitative measure to the qualitative literature data would be helpful. Were these the threshold to determine statistical significance? Please explain.

- The increase/decrease comparison between simulations is explained in the results section under Qualitative validation, as well as in the Figure 2 description "We analyzed model responses to individual stimuli (E2, T, Strain, AngII, and ET-1) relative to baseline, and their combined effects with sex hormones (+E2 or +T) relative to each stimulus (Strain, AngII, ET-1) alone". An absolute difference of 0.05 or above (5% of maximal activity) the steady state value at baseline is chosen as a value to show qualitative effects of the network; this was not chosen to determine statistical significance to literature, but rather to show node behavior in response to upregulation perturbations. This number for thresholding was used in Irons et al. 2021 and we decided to use the same value as we compare a qualitative response of our model to qualitative data in western blots.

4. Ryall, et al [17 in the manuscript] has published a comprehensive model of cardiomyocyte hypertrophy that includes the same pathways as in your model. Does your model behave similarly to the Ryall model, for example if you simulate an increase in the AngII, ET-1, or Strain?

- The Ryall model indeed provided a strong foundation for the cardiomyocyte hypertrophy network model, followed up by Frank et al. When we simulate an increase in Strain, AngII, ET-1 our model agrees with the Ryall and Frank models: in both the models this leads to an increase in CellArea. However, Ryall et al. and Frank et al. have a more exhaustive network of nodes and pathways for cardiac hypertrophy compared to our model, because we only included pathways for which could find experimental evidence that they were mediated by E2 and/or T. For example, our model does not capture the behavior of the BNP pathway. We reflect on this in the Discussion section under Comparison with prior work, lines 268-270.

5. In your qualitative validation, were there any disagreements that stood out? Were these disagreements due to a completely opposite prediction (for example, predicted up-regulation but reported down-regulation), or because the model under or over-predicted a species activity? I would also recommend labeling the boxes in Figure 2 that disagreed with the literature data, especially since there are only 4 of them.

- Thank you for pointing this out. We have added this line in the Results section of Qualitative validation (lines 138-140) to highlight the ones we have missed validating:

  "The literature studies found no change in ERK12 and CellArea due to E2 by itself, but the model predicted a slight decrease. Similarly, we predicted an increase in PI3K due to E2 when literature reported no change. The

model showed no change in PI3K, whereas the data suggests that ET-1 should decrease PI3K while AngII and ET-1 should increase PI3K. Finally, the model did not capture AngII's activation of AMPKf. "

- Per your recommendation, we have also labelled the nodes in Fig 2 that disagreed with literature data with a black box. Additionally, we made some minor adjustments in the model based on reviewer feedback. The new validation analysis indicates that our model correctly captures 32/39 response. The manuscript has been updated accordingly.

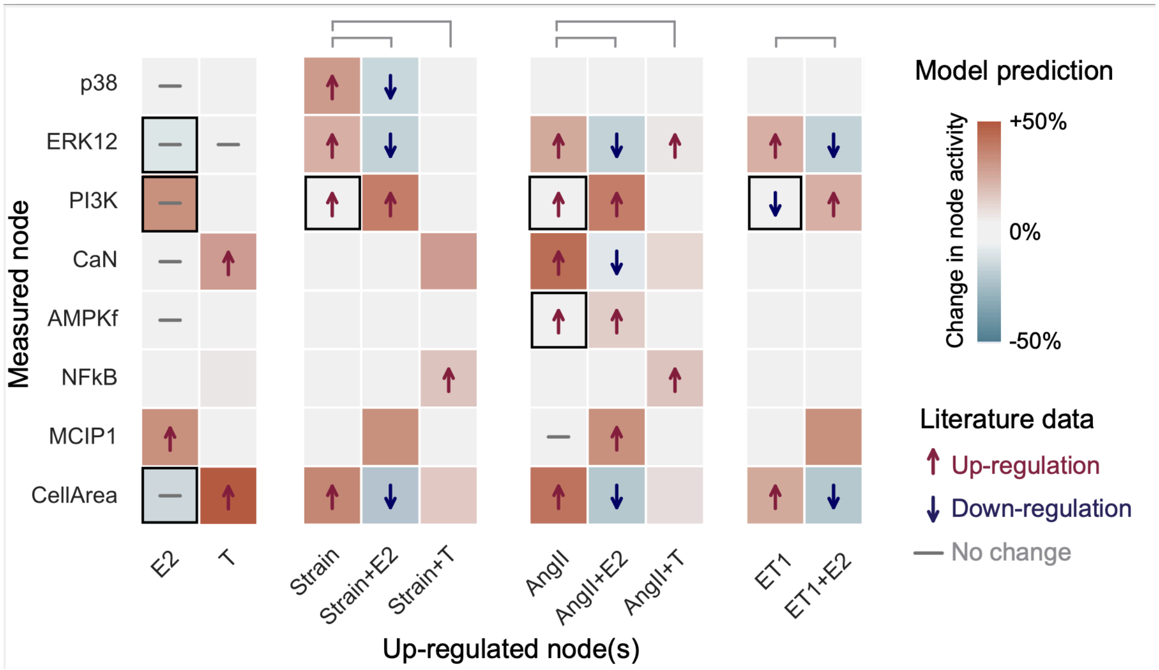

6. "Quantitative Validation" did you look at the steady-state response of the network model? If so, please indicate in the text.

- Thank you for this feedback. We indeed checked if a steady-state was established in this baseline simulation as well in the single perturbation simulations before applying the second perturbation. We now elaborate on this in the text of the Results section on Quantitative validation (Lines 159-161) as:

  "We ensured that a steady-state was established in the baseline simulation and in the single perturbation simulation before applying further perturbations."

7. Quantitative Validation - How did you determine how much to increase each input for these studies? Some details are included in the methods, but the choice seems qualitative without considering how they quantitatively impact CellArea. Is it possible to calibrate the model inputs to quantitatively match any of the data used to build the model?

- We agree with the reviewer that some of the choices we made are semi-qualitative, however we were careful to avoid simply choosing parameter values that would result in predictions that best matched the additional quantitative data, to thus function as true validation. The perturbation increases for the sex hormones were the same as the Δ chosen in the qualitative validation, i.e. 0.3. We set the weight for Strain as 0.6 during the perturbation, based on a previous paper by Yoshida et al. When AngII and ET-1 were added to the serum in in-vitro studies we picked a high weight value of 0.9 to reflect the maximal serum concentrations, while for the in-vivo studies we doubled the baseline values for AngII, to a total of 0.3, as it was not exogenously added or measured. With these weight choices, we have tried to capture real world values that were reported using normalised values between 0-1. Unfortunately, data used to build the model was mostly qualitative, determined through Western Blots. Therefore, it would not have been possible to calibrate model inputs based on the data used to build the model. However, we will keep this idea in mind for future projects where western blots are followed with protein quantification analysis to calibrate our model further.

8. Were the different concentrations of hormones (for example, E2) in the different studies accounted for?

- In quantitative validation (Figure 3) the different concentrations for the hormone inputs were accounted for by modifying the input weights from baseline. For example, as mentioned in the Methods section on Quantitative validation “We adjusted the baseline E2 = 0.5 and an E2 increase of E2 = 0.3 for the in vivo female rats without OVX (ovariectomy) in Donaldson et al. and baseline E2 = 0.01 and an E2 increase of E2 = 0.3 for the in vivo female rats with OVX in (Chen et al. 2022)”, and, “E2 and T perturbations were simulated by setting E2 = 0.5 and T = 0.21 for the in vitro studies.” This explains that for the Donaldson study in intact females the baseline E2 weight was set to 0.5, in the Chen study with OVX females the baseline E2 weight was set to 0.01, while for the Pedram study the baseline E2 weight was set to the calibrated input baseline. The perturbation changes for all the in-vivo models was 0.3 which was the value used for perturbations in the qualitative validation (Figure 2). The modified E2 weight for the in-vitro model was set to 0.5 to reflect a mixed pool of pregnant and neonatal rats, which aligns with how we calibrated for the pre-menopausal female in the Methods section of Model Calibration: “For males, we set $w_T=0.21$ to achieve a CellArea of 0.65, reflecting the post-pubertal 1.3-fold increase in male left ventricular wall mass relative to females Simone et al. Similarly, for post-menopausal females we set $w_{E2}=0.01$ to achieve a CellArea of 0.54, reflecting the post-menopausal 1.08-fold increase in female left ventricular wall mass relative to pre-menopause Appiah et al. Since there was no clear developmental stage to calibrate the increase of E2 in pre-menopausal women, we chose to set $w_{E2}=0.5$, which is in the center of its range, leaving space for

higher degrees of up-regulation, such as during pregnancy and the ovulation phase of the menstrual cycle and pregnancy."

9. AngII is known to increase in response to aortic banding. Was this increase accounted for in the Donaldson study?

- Thank you for pointing this out. We had not modified AngII in the Donaldson study in response to Aortic banding originally, but we have now made this modification wherein both Strain and AngII are modified. We have now included the % change in CellArea values in the text, and made modifications in the rest of the manuscript while talking about the in-vivo quantitative validation studies results as follows, in section methods under model validation, lines 498-507:

  "As AngII is known to increase in response to aortic banding, we increased w_AngII by a factor two of its baseline value, as it was not exogenously added or measured."
- And in the results section under quantitative validation, lines 153-156:

  " For the in vivo studies, we overestimated the impact of strain: our model predicted a 152% change of CellArea compared to the observed 41.7±3.25 % change induced by aortic banding in female rats by Donaldson et al., and 32.7% vs. 21±2.15% for its mitigation by E2 (Fig. 3b)."

10. Similarly, was strain increased in the spontaneously hypertensive rat study? This was not clear from the text. Were these studies in male or female rats? Also, which bars in Fig.3b refer to the Donaldson vs. Chen data?

- Thank you for pointing this out. We had not modified Strain in the Chen study for spontaneously hypertensive rats, but we have now made this modification wherein both Strain and AngII are modified. Both the studies (Chen et al. and Donaldson et al.) were in female rats. We have now made modifications in the rest of the manuscript while talking about the in-vivo quantitative validation studies results as follows, in section methods under model validation, lines 498-507:

  "As Strain is known to increase in response to spontaneous hypertension, we set w_Strain = 0.6."
- And in the results section under quantitative validation lines lines 156-158**:**

  "For a spontaneous hypertensive rat model from Chen et al., we under-estimated the anti-hypertrophic effect of E2 (-5\% predicted vs. reported -14±1% change in CellArea) and underestimated the combined effect of E2 and T (-2 predicted \% vs. reported 19±1.25% change in CellArea)."

- The changed figure (b) with the changes in comments accounted for looks like this:

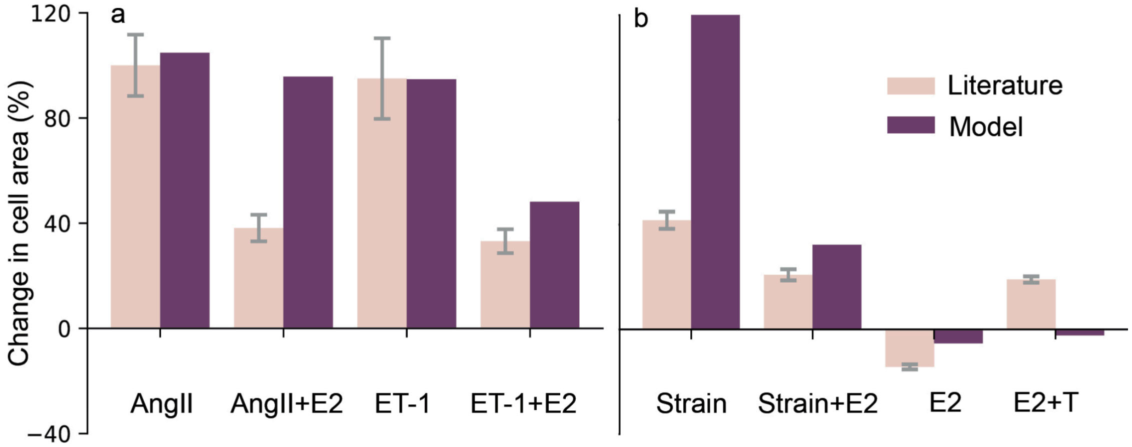

11. One concern is that we wouldn't expect a significant change in "Strain" in response to aortic banding, but instead an increase in stress. I would be careful with the wording to emphasize that this is a generic mechanical input (earlier on in the manuscript)

- Since strain and stress are constitutively related, we expect that aortic banding increases both strain and stress. However, we do agree with the reviewer that it is unclear whether cells transduce strain or stress into hypertrophic stimuli. Lines 95-97 in the Results section under network structure now reflect this:

  " Strain and stress are constitutively related and we expect both to increase during aortic banding, however we refer to mechanical change due to pressure overload as a composite Strain input in our model."

12. Cross-talk between hypertrophic stimuli - - Were the in vivo studies in ovariectomized or intact females? Were the expected concentrations of E2 accounted for to reflect this?

- In the quantitative validation section, the in-vivo study by Chen et al. was in OVX animals while the in-vivo study by Donaldson et al. was in intact female animals. The baseline E2 weights were indeed adjusted to reflect this, and was covered in the Methods section of the manuscript as well as comment 8 above. In the "cross-talk between hypertrophic stimuli" section of the Results (Figure 4) we explore how the model inputs together affect cell area, essentially showing the generic pro-hypertrophic effects of Strain, AngII, T and anti-hypertrophic effects of E2 on CellArea. This shows how the model output changes for the whole range of input values from 0-1, not just for certain concentration ranges.

13. Case Studies: comparisons between sex and age - - Did you validate or is it possible to validate some of these cross-talk predictions?

- We have not attempted to validate the case study predictions between sex and age specifically. However, because of the earlier validation of the effect of E2 and T (with and without the other stimuli), we are confident that these predictions correctly describe differences in signaling between the sex and age groups. Further, we did not come across any dose-response curves for both sex hormones on the same downstream nodes which would be required for this validation. However, we will keep this suggestion in mind for future work, especially when we deal with specific hormone concentrations and drug effects for patients.

14. I had a hard time understanding the Influence and Sensitivity analysis in Figure 5, as well as the description in the text. I would recommend re-organizing this section. For example, focusing on the influence first, then sensitivity after, etc.

- We apologize for the confusion while reading the Results section of Case studies lines 195-204. This section previously read as

  "During up-regulation, T is found to be more influential than all other nodes in all three models (Figure 5a). CellArea is the most sensitive node during this up-regulation, which is expected since it is the most downstream node. While the post-menopausal and male models have similar influence and sensitivity scores, there are differences in the pre-menopausal model. During knock-down analysis(Figure 5b), intriguingly, endothelial nitric oxide synthase (eNOS) becomes the most influential node for all three conditions, while T is the second most influential node in males only. CellArea continues to be a highly sensitive node for all three models in this knock-down scenario. The increase in Akt's sensitivity in the pre-menopausal condition might be significant for hypertrophic regulation."

  and was restructured as suggested by the reviewer to the following:

  "During up-regulation, T is found to be more influential than all other nodes in all three models (Fig 6a). During knock-down analysis (Fig 6b), intriguingly, endothelial nitric oxide synthase (eNOS) becomes the most influential node for all three conditions, while T is the second most influential node in males only. We also note a slightly higher influence of cGMP in post-menopausal females during knock-down. CellArea is the most sensitive node during up- and down-regulation for all 3 models, except for PKG1 being more sensitive than CellArea in males during knock-down. While the post-menopausal and male models have similar influence and sensitivity scores, there are differences in the pre-menopausal model. The increase in Akt's sensitivity in the pre-menopausal condition might be significant for hypertrophic regulation."

15. What does it mean biologically that T is most influential? I understand that computationally, up-regulating T will influence the activity of all other species the most. Does this mean that you would expect Testosterone to have a largest effect on cardiac hypertrophy compared to other inputs/species? Please explain the significance of this analysis.

- Thank you for this feedback. We indeed expect that T will have a larger effect on cardiomyocyte hypertrophy than any of the other input species, based on the combined results of the case studies and sensitivity/influence analysis. In the model, that means a certain change in T will cause a bigger change in CellArea than the same change in any other input. Assuming that the normalized model values of the inputs span a physiological range, that means that biologically, physiological changes in T will have a larger impact on cardiac hypertrophy than changes in other inputs. This can for example be observed during puberty, where T increases in boys and E2 in girls, yet only the heart size of boys deviates from the linear growth curve observed up to that point. We have added a line to talk about this in the discussion section under Physiological implication, lines 315-322:

  "Based on the combined results of the influence/sensitivity analysis and case studies, we expect that T will have a larger effect on cardiomyocyte hypertrophy than other input species. In the model this implies that a certain change in T will cause a bigger change in CellArea than the same change in other inputs. Biologically this implies that physiological changes in T will have a larger impact on cardiac hypertrophy than the other inputs, as for example observed in puberty where T increases in boys and E2 in girls, yet only the heart size of boys deviates from the linear growth curve observed up to that point. "

16. "T's higher influence when compared to E2 suggested by this model is important in explaining the differential response to cardiac hypertrophy in pre-menopausal females, post-menopausal females and males, as well individual differences within these categories."What does this mean? What data do you have to support that T has a higher influence compared to E2 clinically?

- T being the most influential node in our model might explain differential response to cardiac hypertrophy in pre-menopausal females, post-menopausal females and males, as well individual differences within these categories. This is because, E2 by itself cannot explain the hypertrophy differences between post-menopausal females and age-matched males as these two groups have the same amount of estradiol. This hypothesis suggested by our model is further supported by a recent study by Methawasin et al where testosterone, and not just estrogen deficiency, is proposed to drive HFpEF susceptibility and hypertrophy in an ovary-intact post-menopausal female mice model. This has been added to the Results section under Case Studies, Lines 205-215:

  > "T being the most influential node in our model might explain differential cardiac hypertrophy response in pre-menopausal females, post-menopausal females and males, as well individual differences within these categories. E2 by itself cannot explain the hypertrophy differences

between post-menopausal females and age-matched males as these two groups have the same amount of estradiol. This model finding is supported by a recent study by Methawasin et al. where testosterone, and not just estrogen deficiency, is proposed to drive HFpEF susceptibility and hypertrophy in an ovary-intact post-menopausal female mice model."

17. Discussion: An important implication of your model could be explaining why hormone replacement therapies are controversial for cardiac health in post-menopausal women (https://www.physiology.org/doi/10.1152/ajpheart.00041.2018) or possibly for gender affirming care. Are there any insights gained from your model for informing/explaining the effects of hormone replacement therapies?

- Thank you for this excellent point. We are currently pursuing this direction and have added this line to the manuscript in the Discussion section under Future Considerations, Lines 421-426:

  "In the future, we see our model being used to answer questions such as understanding how hormone replacement therapy (HRT) impacts cardiac remodeling in post-menopausal females, and why E2 supplementation to pre-menopausal levels does not improve health outcomes (Speth et al 2018). We would also like to use our model to understand the unknown outcomes of gender affirming hormone therapy (GAHT) on the heart (Masumori and nakatsuka 2023)."

Reviewer 3

1. In lines 86-87 ("We opted for a single composite output, CellArea...") while this makes sense in order to have a directly quantifiable output for what you wish to measure - increase or decrease of cell size - it does obscure a bit the network and how the ODEs apply to it. This is because now the nodes do not represent only biomolecules (line 73). The same occurs with having Strain as an input node. In particular, it is not entirely clear to me what a CellArea = 0 means (or Strain = 1). Neither is the physical mechanism for the nodes to "interact" with CellArea, i.e. Hill Functions are usually explained in terms of the concentration levels of the biomolecules with the function parameters representing the different physical interactions between them (e.g. binding). This needs to be directly addressed in the main text.

- We thank the reviewer for evaluating our manuscript and providing constructive comments. We agree with the reviewer that the CellArea and Strain nodes are simplified representations of the more nuanced changes in biomolecules. Ideally, we would have biomolecules such as actin and myosin in our network that together contribute to the change in cell size, but different ratios would also have implications for cellular behavior and function, for example contractility.Therefore, to reduce model complexity and remain focused on hypertrophy, our current model is limited to predicting a composite response in CellArea. Similar for Strain, we could not find studies on the impact of sex hormones

on hypertrophy-related integrins and ion channels that together sense and translate changes in the cell's mechanical environment.

- As for the model values of Strain and CellArea, because of the normalized Hill functions these represent normalized concentration levels, as the reviewer suggested. So a value of 0 means Strain has no effect (inhibition nor activation) on downstream reactions, while a value of 1 means a maximum effect. For CellArea, we adopted the approach from previous studies (Yoshida et al., Estrada et al.) and considered a CellArea of 0.5 the steady-state value, with 0 representing the lowest possible cell size and 1 representing the maximum possible cell size.

    - We have made the following changes in the manuscript to better reflect these choices and limitations:

        We have added this line to the discussion section under limitations, lines 390-396:
        "In our model CellArea is a simplified representation of more nuanced changes in biomolecules, such as actin and myosin, However since their ratios would have implications for cellular behavior and function such as contractility, we did not include them in our current model of hypertrophy. For Strain, a composite input, we could not find studies on the impact of sex hormones on hypertrophy-related integrins and ion channels that together sense and translate changes in the cell's mechanical environment."

        We modified Line 73 (Line 77) in revised manuscript) to say the network is not just biomolecules *…"generic input and output (Strain and CellArea)"*

        In methods, section on logic-based normalised hill type equations, we have added this Lines 447-451: *"For the generic composite nodes that aren't biomolecules such as Strain which act on several molecules, a value of 0 means Strain has no effect on downstream reactions, while a value of 1 means a maximum effect. For the generic output node, CellArea, a value of 0.5 represents the steady-state baseline, with 0 representing the lowest possible cell size and 1 representing the maximum possible cell size"*

2. In lines 80-81, as well as 316-317, it is explained that because E2 and T "are linked to a similar number of downstream nodes" there is no connectivity bias as to their downstream influence. However the degree of a node alone (or even the number of nodes in its downstream paths - which is what you seem to be referring to) does not determine the influence or centrality of a node. Two nodes A and B can be connected to the same amount of nodes but if node A's neighbors lead to nowhere whereas B's neighbors do, then it follows that node B will have a bigger influence in network dynamics. The focus should be the different pathways from the input

nodes to the output node CellArea. I recommend to include a simple analysis of node centrality or influential nodes in the network. This can also be a useful comparison to results in Fig 5. By looking at Fig 1 it seems that for the most part T and E2 downstream paths rarely cross, except at nodes eNOS and ERK12. eNOS seems to act as a "bottle-neck" for multiple pathways from E2 and one pathway from T, which probably explains why it is such an influential node under knock-down analysis (lines 176-178).

- Thank you for pointing us in this direction and thinking about centrality. We had calculated connectivity as counting all the descendents from each input. When first calculating centrality we identified all descendants from inputs that go to the output (27 for E2, 24 for T), and the paths for each input leading to the output (16 for E2 and 15 for T). This made sense since there is only one output in our model. Then, we checked for 'betweenness' or bottleneck on paths to outputs which is defined as how often a node lies on the shortest path thus making it influential. The value for betweenness was 0.0 for inputs as expected, the top 3 species that are bottlenecks in the network are ERK12 (0.028), eNOS (0.025), and NFAT (0.020).
- Based on the reviewer's suggestion, the Results section under Network Structure, lines 83-86 was changed from describing *"In the resulting network, E2 and T are connected to a similar amount of downstream nodes, 27 and 24, respectively"* to :

  "*In the resulting network, E2 and T are connected to a similar amount of downstream nodes, 27 and 24, respectively. We additionally assessed bottlenecks in our network, defined as how often a node lies on the shortest path thus making it influential. The top 3 bottlenecks in our network were ERK12, eNOS and NFAT.*"

3. Line 75, as well as 395, mention the use of OR gates but Fig 1 only shows AND gates. Additionally, how do you account for more than 2 inputs received? As in node MEF2 receiving 3 inputs, or even CellArea? Please address this explicitly.

- We apologize for the lack of clarity in Fig. 1. For readability (similar to prior logic-based network studies) we choose to not explicitly show OR gates, instead only showing AND gates. OR gates are present when more than 1 node influences a downstream node. We have now explained this approach in the methods section. "In Fig 2 the AND gates are shown by '&' and OR gates exist when more than 1 node influences a downstream node.", and the caption of Figure 1: "AND gates are shown by \&' and OR gates exist when more than 1 node influences a downstream node."
- Additionally, we have shown how a change of activity in a node with more than 2 inputs (including AND and OR gates) is calculated in Equation 8 of the original submission.

4. Fig 1 suggestions: I think the figure would be improved if you make visually clear the top-down structure of the network, by putting all input nodes atop, with the intermediate nodes strictly beneath them. Additionally, you can just use a smaller, differently shaped node to stand

for AND gates and add it to the legend. This way you avoid the need to put the word AND so many repeated times, removing some visual clutter. Preferably I would also use a different shape for CellArea since it is not an actual biomolecule as the rest of the nodes but more of a standing for the network's output

- Thank you for these suggestions, we changed the network visualization as the reviewer suggested and agree that Figure 1 is now much improved after taking into account these suggestions.

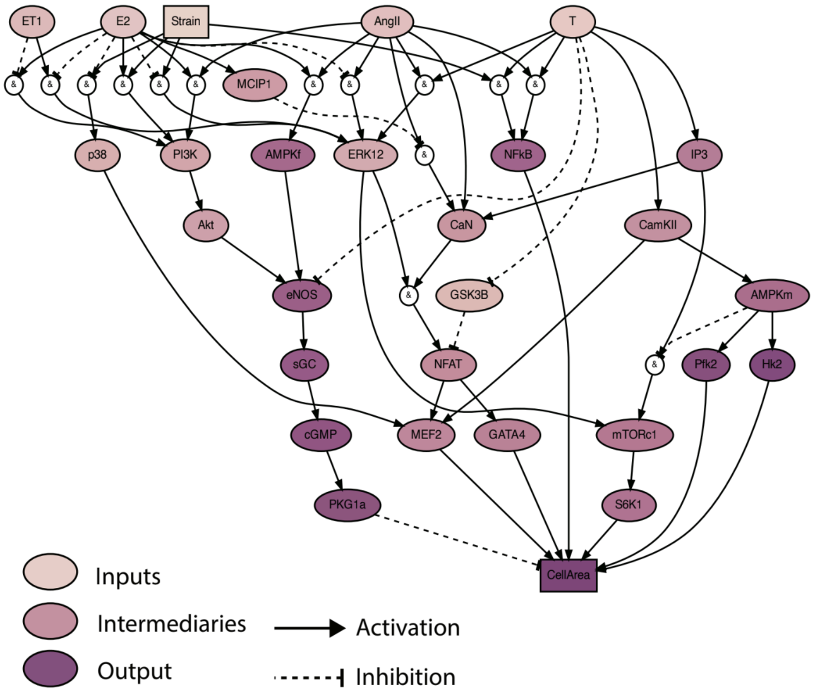

5. In lines 96-98 the conflicting behaviour of this specific path by sex is mentioned, could you give a plausible explanation as to how this is the case? Perhaps there is an intermediate node modulating this different behaviour or something similarly Explainable?

- We have also been curious about this change between the sexes, and hope that this paper makes a case for focused experimental work to understand sex differences where all the pathways are investigated at the same time in the same experiment.
- We performed additional research and corrected a reaction in our model discussed in these lines, where instead of AMPKm inhibiting eNOS it is now T inhibiting eNOS as the E2/eNOS pathway is not activated in males (Sasak et al.). This sex difference is hypothesized to be due to a couple of potential reasons. It could be due to VEGF's role in males, differences in cav-3 protein which plays a big role in eNOS activation, or, the number of ER-b between the sexes. (Sasak et al.
- Additionally, the decision to split AMPK into AMPKm and AMPKf is because we had literature experiments done in those specific sexes.

- In general, we see that there is also a need to better understand the PI3K pathway, and when the switch between physiological and pathological states happens, and especially between the sexes.
- This has been added in Results - Network Structure lines 105-112 as:

  "The second exception is where the influence of T on eNOS was inferred, as the E2/eNOS activation is seen in females, but not in males (Sasaki et al.). This sex difference is hypothesized to be due to a couple of potential reasons. It could be due to VEGF's role in males, differences in Cav-3 protein which plays a big role in eNOS activation, or, the number of ER-b between the sexes. Additionally, the decision to split AMPK into AMPKm and AMPKf is because we had literature experiments done in those specific sexes. This separation thus allows for a more nuanced expression of the sex-specific effects on AMPK signaling in the model."

6. In section Quantitative validation (line 125), you write 'under-predicted', 'overestimated', etc.., but since this is easy to see from Fig 3, perhaps it would be helpful if in the text you write by how much you under/over-estimated instead, which is harder to see precisely from the fig

- Thank you for your suggestion, that is indeed more useful, we have now added the values where we discuss the results under the Quantitative validation in Lines 149-158 as follows:

  "We correctly matched increases in CellArea due to AngII and ET-1. We overestimated AngII mitigation by E2 (95.7% predicted vs. reported 38±5% change in CellArea), and overestimated E2's mitigation with ET1 (48% predicted vs. reported 33±4.5% change in CellArea)."

  For the in vivo studies, we overestimated the impact of strain: our model predicted a 152% change of CellArea compared to the observed 41.7±3.25 % change induced by aortic banding in female rats by Donaldson et al., and 32.7% vs. 21±2.15% for its mitigation by E2 (Fig. 3b). "For a spontaneous hypertensive rat model from Chen et al., we under-estimated the anti-hypertrophic effect of E2 (-5\% predicted vs. reported -14±1% change in CellArea) and underestimated the combined effect of E2 and T (-2 \% predicted vs. reported 19±1.25% change in CellArea)."

7. Lines 173-174 mention that CellArea is expected to be the most sensitive node since it is the most downstream node. But this implies that the network is not robust at all with respect to changes in the input/intermediate nodes. Is this biologically desirable? Shouldn't it be more advantageous for the network to be robust to changes so that perturbations don't result in hypertrophy?

- This is a valid point that was not addressed in the initial manuscript. We agree that it is not desirable for CellArea to be so sensitive to changes in the inputs. We believe this is the consequence of three limitations in our model. First, we have only one composite output, as discussed earlier. So more nuanced signaling

pathways pertaining to synthesis and degradation of various biomolecules, such as in Irons et al. for vascular cells, could lead to a more robust behavior. Second, there are no feedback mechanisms in our network that could stabilize the model. For example, in the multiscale model formulation in Estrada et al., an increase in CellArea leads to a reduction in Strain because of tissue thickening, thus stabilizing cardiomyocyte hypertrophy. Third, our calibrated model inputs are situated on the steeper part of the Hill curves, causing any perturbation to have a relatively large downstream effect. We now include the following lines to address this in our manuscript in the discussion section under limitations, lines 381-396:

> "Fourth, the CellArea output in our model is very sensitive to input changes. We believe that this is a consequence of having one single composite output for the model. So more nuanced signaling pathways pertaining to synthesis and degradation of various biomolecules could lead to more robust behavior. It could also be due to a lack of feedback loops to stabilize the output. For example, in the multiscale model formulation by Estrada et al. discussed above, an increase in CellArea leads to a reduction in Strain because of tissue thickening, thus stabilizing cardiomyocyte hypertrophy. Our calibrated model inputs are situated on the steeper part of the Hill curves, causing any perturbation to have a relatively large downstream effect, thereby increasing the sensitivity of our output."

8. Line 205, "exceeds" but barely so, from figure 6 it looks almost the same value.

- We have modified Line 231 of the Results section on Case studies:

  > "Interestingly, the post-menopausal model (low E2) initially starts close to the pre-menopausal curve but eventually (at Strain > 0.5) matches the male curve, indicating that this model did not have sufficient E2 to prevent hypertrophy at high Strain levels when compared to the pre-menopausal female model."

9. Line 279 "we can attribute to increased E2" sounds off, perhaps add levels: "we can attribute to increased levels of E2".

- Thank you, due to other changes this line has been removed from the manuscript.

10. Lines 334-337 and 353 talk about the need and impact of including organ-scale models. Could you briefly elaborate how you could extend your model to include These?

- We thank the reviewer for this question. We indeed talk about coupling cell-level network models to organ-scale models in the Discussion - Limitations and Future considerations sections. Coupling can be achieved through the kinematic growth (as in Estrada, Yoshida, Kaissar) or the constrained mixture method (as in Irons), where the cell-level model drives changes in tissue mass and/or composition in the organ-level model.

11. Equations 9 and 10 in subsection Influence and sensitivity analysis correspond to two different summations. The variable delta y should actually include two indices: one for the node (the only one explicitly shown) and another one for the case (i.e. different knockdown/upregulation instances). This way the influence of a node is a summation over all nodes (for a given case), while the sensitivity is the summation over all the cases (for that given node).I.e. (9) = Sum over i for all y_ij while (10) = Sum over j for all y_ij; i stands for the node and j for the knockdown/upregulation case

- Thank you for pointing this out to us, equations 9 and 10 have been changed to show y_ij in the revised manuscript.
- We also had typos in the sentence describing Sensitivity before the equation for the same, this has been rectified in the methods section under Influence and Sensitivity analysis**,** lines 519-520:
  "Conversely, sensitivity S_i of species i was defined as the sum of absolute change in activity of the species across knockdown/up-regulation of all other species j: "

Reviewer 4

1. Lack of formal sensitivity analysis (e.g., Sobol's indices) to assess uncertainties in the newly added network nodes. Such an analysis would clarify the most influential nodes and pathways mediating sex hormone effects in male and female hearts, a standard practice in prior studies. This approach could uncover key nodal signals and clustered pathways critical to hypertrophy, deepening mechanistic insights.

- We thank the reviewer for evaluating our manuscript and providing constructive comments. We agree with the reviewer that additional sensitivity analysis is insightful so we added a global parameter sensitivity analysis using Sobol's method. This addition is present as Figure 5 (a,b) in the new manuscript before the local influence and sensitivity analysis. Note that Sobol's method, like most global sensitivity analyses, assumes that inputs are independent. Because several downstream nodes are dependent on each other through interconnected signaling pathways, we only included the most upstream nodes as inputs in the Sobol analysis.

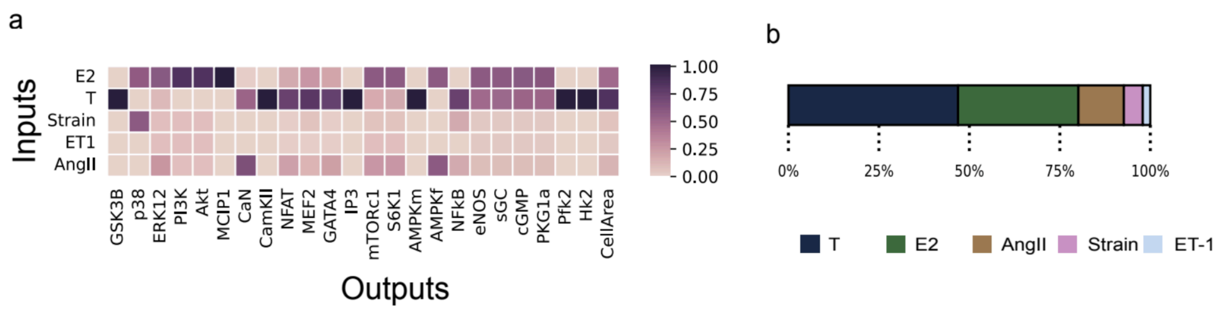

- The results showed that T and E2 have a higher influence on many downstream nodes when compared to other inputs, with T being more influential on CellArea than E2. We can also see which specific pathways are influenced to a greater extent by Strain, AngII and ET-1, which will be important when understanding the influence of drugs on specific pathways in the future. This has now been added to the results section under Cross-talk Lines 176-180 of the revised manuscript as :

  > "Conducting a global sensitivity analysis using Sobol's method, we observe T and E2 to have a higher influence on many downstream nodes when compared to other inputs, with T being more influential on CellArea than E2. We also see which nodes are influenced to a greater extent by each of the inputs,, which will be important when understanding the influence of drugs on specific pathways in the future."

2. Interpretation of hormone impact comparisons—The model's major novel finding is that testosterone has a greater effect than estradiol on hypertrophy. However, simulations rely on normalized upregulations/knockdowns rather than exact hormone concentrations or nodal expression levels. Thus, the physiological relevance of these comparisons remains unclear.

- We acknowledge that our model uses normalized concentrations or nodal expression levels. In the future we wish to couple this network model to an organ-level model (as described in Reviewer 3 comment #10 and in the future consideration section of discussion). This multi-scale model will incorporate physiological levels of sex hormones, as well as cell area change that is translated to myocardial mass change. We can convert physiological hormone concentration values into normalized values using linear or non-linear transfer functions. With this multi-scale formulation we hope to have more physiological relevance in observing remodeling differences between pre- and post-menopausal females and males.

3. Aging considerations—Aging (e.g., pre- vs. post-menopausal females, males) is modeled solely by adjusting sex hormone levels. Yet, aging involves complex changes in multiple nodal signals within the network. The extent to which this simplification reflects real-world cardiac aging is uncertain. Given that cardiac hypertrophy is age-associated—and aging reduces testosterone in males—the model's ability to explain this observation warrants further exploration.

- We agree with the reviewer that the way we simulated aging is simplified and that nodes other than E2 would need to be adjusted. We chose to only consider a decrease in E2 in females. We chose to not explore age-related decreases in T in males since this change is much smaller (~10%) compared to the decrease of E2 in females (~60%) from 45-65 years of age when transitioning to menopause (Dorak 2017). We have now commented on this in the Discussion section under Physiological considerations, Lines 322-326:

  > "We chose to only consider a decrease in E2 in females and not explore age-related decreases in T in males since this change is much smaller

(around 10%) compared to the decrease of E2 in females (around 60%) from 45 to 65 years of age while transitioning to menopause (Dorak 2017). "

4. Potential therapeutic targets—As noted, the manuscript could be enhanced by investigating druggable targets to mitigate hypertrophy in aged males and females, offering additional translational relevance.

- We agree with the reviewer and wish to do this in the future with a sex hormone network model that also accounts for contractility. Even with the current model for hypertrophy, we can simulate the effect of (1) ARBs and beta-blockers in aged males and females to show the difference in remodeling and (2) simulate the effect of drug valsartan/ sacubitril. The PARAGON ARNI trial with the drug valsartan/ sacubitril targeting the AT1R and eNOS/cGMP pathway respectively almost showed sex difference favorable to females for HF, and was short by a very small p-value difference (McMurray et al 2020).

When revising the manuscript following the reviewer comments, we made some minor additional changes:

- There was a typo in the Methods section under Logic-based normalised Hill equations: where MCIP1 was written instead of IP3 to describe equation 7 which has been changed to IP3:

  "Using the normalized Hill functions, the change in activation of IP3 is defined as"
- We corrected a typo in parameter simulations, and one model reaction, which lead to the following changes:
  - The baseline weight for all inputs changed from 0.22 to 0.15 in the methods section of model calibration:

    Lines 474: "Multiple parameter combinations could achieve this equilibrium, as seen in Fig 4, so we performed the calibration by constraining all inputs to a single value, which converged to w_E2=w_T=w_AngII=w_ET1=w_Strain= 0.15."
  - Because we added a reaction based on a reviewer comment, we had to repeat the baseline calibration. The values for w_T=0.29 for males and w_E2 for post-menopausal females in the calibration section of methods have changed to 0.21 and 0.01 respectively:
    - Lines 476: "For males, we set w_T=0.21 to achieve a CellArea of 0.65, reflecting the post-pubertal 1.3-fold increase in male left ventricular wall mass relative to females (Simone et al.). Similarly, for post-menopausal females we set w_E2 = 0.01 to achieve a CellArea of 0.54, reflecting the post-menopausal 1.08-fold increase in female left ventricular wall mass relative to pre-menopause (Appiah et al).
    - In model validation reflect the same change as above, lines 505: "E2 and T perturbations were simulated by setting w_E2= 0.5 and w_T = 0.21 for the in vitro studies" and lines 510: "...baseline w_E2 = 0.01 and an E2 increase of 0.3 for the in vivo female rats with OVX in Chen et al."
