# Supplementary material for: Modeling the interplay of sex hormones in cardiac hypertrophic signaling
Source: PLoS Comput Biol. 2025 Dec 10;21(12):e1012895. doi: 10.1371/journal.pcbi.1012895 (PMC12704863; doi:10.1371/journal.pcbi.1012895)
Supplement: Attachment — Submitted filename: AKL_PJO_Paper1_2nd_rebuttal_2025.pdf [file pcbi.1012895.s005.pdf]

Response To Reviewers

PCOMPBIOL-D-25-00341
Modeling the Interplay of Sex Hormones in Cardiac Hypertrophic Signaling
PLOS Computational Biology

We would like to thank all reviewers for their rigorous and meaningful feedback, which we believe has strengthened our manuscript.

We have addressed Reviewer 4’s remaining feedback and request for simulations in this rebuttal document.

Reviewer 4
1. Aging and sex hormone levels - "We chose to not explore age-related decreases in T in males since this change is much smaller (~10%)". Additional simulations with 10%-20% reduction with aging would be essential to help consolidate the claimed conclusions. This is important because the sensitivity analysis does indicate a strong influence of T on the investigated components related to hypertrophy.

- We thank the reviewer for evaluating our manuscript and providing constructive comments. In our original manuscript under Methods (section: calibration), we had set the value of T for “younger” males such that it leads to an increase in CellArea from 0.5 to 0.65 to match data from Simone et al. Per the feedback from the reviewer, we now added an additional model called “older male” to explore the impact of a reduction in T by 20%. This resulted in a CellArea of 0.53, so reduced compared to the younger male but higher than the baseline of 0.5. We made the following observations from the older male model simulations:
    - With the influence and sensitivity plots, the post-menopausal female, older male and male models have similar influence and sensitivity behavior.
    - During up-regulation, we noticed that the magnitude of T influence and the CellArea sensitivity of the older male model was more similar to the post-menopausal model than to the younger male model.
    - During knock-down, we notice that the magnitude of eNOS influence and the magnitude of CellArea sensitivity of the older male model was similar to the post-menopausal model. For both analyses the magnitude of CellArea was higher in older male and post-menopausal female models when compared to the younger male model.
    - We have added this to Fig 6 comparing pre-menopausal female, post-menopausal female, younger male and older male models. We added lines 219-225 in Results, Case studies to reflect this:
      “In the older male model with 20% reduced T, the influence of E2 during up-regulation analysis decreases when compared to a male with higher T or a post-menopausal female model, as there is lesser hypertrophy present (Fig 6). We also notice the magnitude of CellArea sensitivity in the older male model

during upregulation and knock-down analysis to be similar to the post-menopausal females. The post-menopausal, male and older male models behave more similarly to one another."

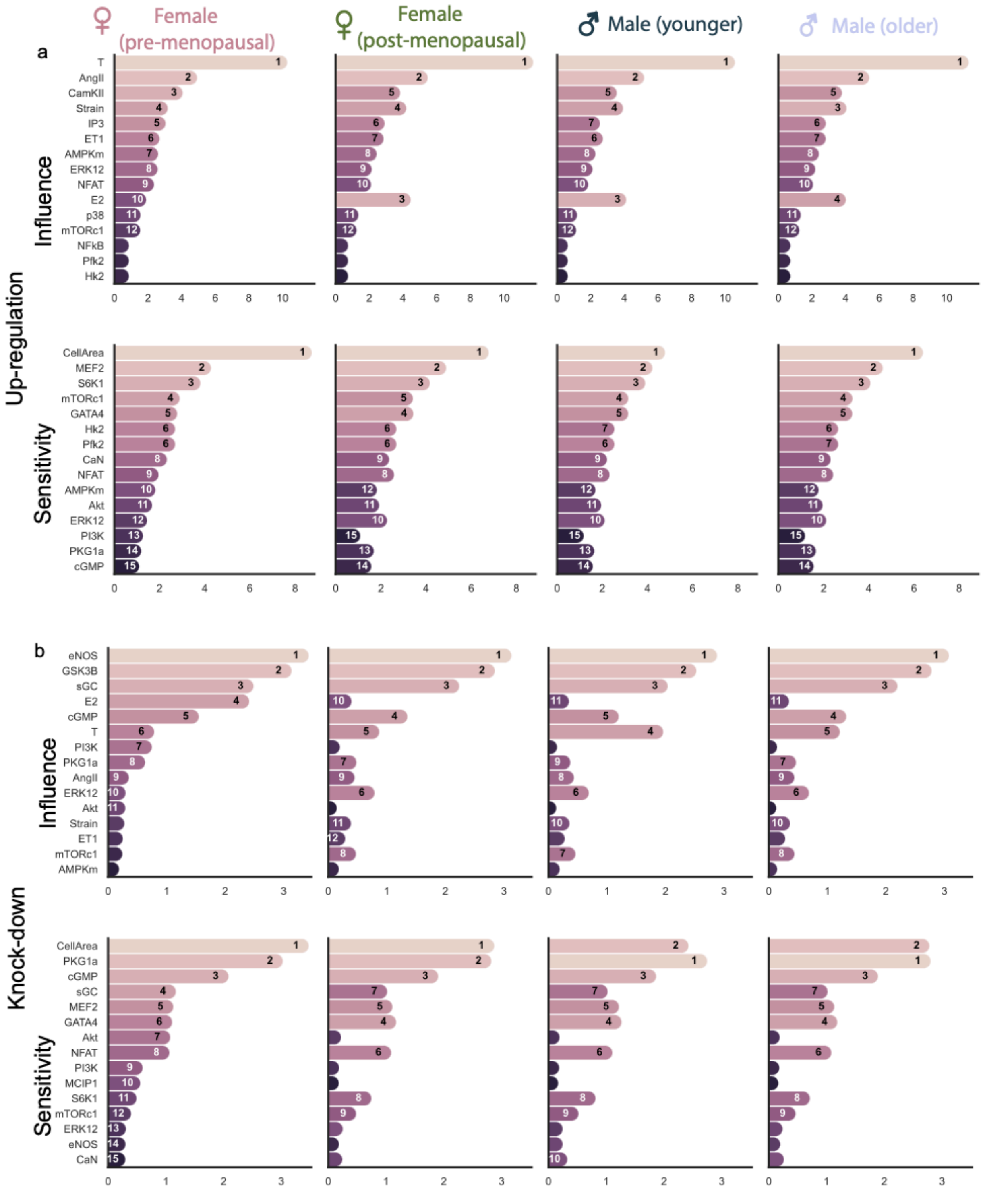

○ With the line plots showing CellArea behavior for different inputs and intermediates, we noticed that the older male model seems closer to the

postmenopausal model than younger male model for specific nodes at low concentrations (T, Strain, AngII, ET-1, CamKII) or higher concentrations (eNOS, GSK3B).. We added lines 247-249 in Results, Case studies to reflect this:

"The older male model with 20% reduced T is closer to the post-menopausal model than the younger male model for specific nodes at low concentrations (in the case of T, Strain, AngII, ET-1, CamKII), or higher concentrations (in the case of eNOS and GSK3B) (Fig 7)."

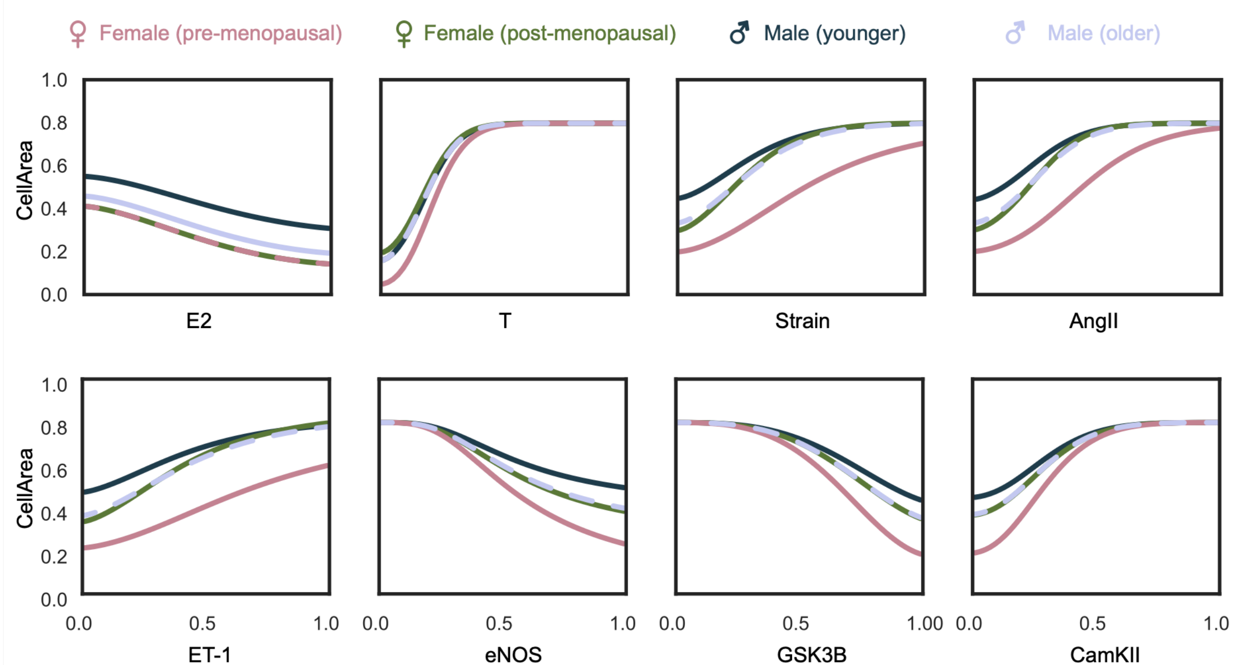

- Further, we have added the older male category in other areas of the manuscript wherever we discuss the other 3 categories (Abstract, Author Summary, Results (Case Studies), Discussion (Physiological Implications section), and Methods (Model Calibration).

2. Potential therapeutic targets - the authors describe compounds "valsartan/ sacubitril" in the responses. It would be valuable without requiring excessive effort to simulate the effects of these drugs using the model.

- We agree with the reviewer that our model can be used to simulate the effect of these drugs and are excited to use it for that purpose. We are working on a separate manuscript to rigorously assess how multiple cardiovascular drugs interact with sex hormones. Because many drugs also impact contractility, we are working on an expansion of our network model to better capture these drug effects. Since the current manuscript is focused on hypertrophy, we consider these simulations to be outside the scope of the current work.

3. Since the aging model appears to lack detailed descriptions of aging-related changes in the cardiomyocytes, it is recommended to tone down the description of aging model in the manuscript, and replacement by 'aging-like' would be more faithful.

- We agree with the reviewer that we model a simplified version of aging and have modified lines 244-245 in the Results>Case studies Section and lines 257-259 in Discussion Section to highlight this:

  "Altogether, these predictions underscore the model's potential to predict age-like and sex-specific hypertrophic responses to combinations of various stimuli."

  "Our sensitivity and influence analyses suggest that differences in T may be more important than E2 in explaining age-like and sex-specific differences in susceptibility to hypertrophic cardiomyopathy."
