# Supplementary material for: Modeling the interplay of sex hormones in cardiac hypertrophic signaling
Source: PLoS Comput Biol. 2025 Dec 10;21(12):e1012895. doi: 10.1371/journal.pcbi.1012895 (PMC12704863; doi:10.1371/journal.pcbi.1012895)
Supplement: Attachment — Submitted filename: PLOSCompBio_Review_April2025.docx [file pcbi.1012895.s001.docx]

**Reviewer:**

**Summary:**

The manuscript by Lakshmikanthan et al. effectively uses network-based modeling and logic-based systems biology to investigate the role of sex hormones in the development of cardiac hypertrophy. By focusing specifically on the influence of testosterone (T) and estradiol (E2), the authors fill an important gap in the existing literature, offering new insights into how sex hormones may modulate cellular behaviors associated with hypertrophy. The authors employ a rigorous methodology, including perturbation, validation, and sensitivity analyses, ensuring that the model is both robust and comprehensive. These analyses address critical aspects of the work, confirming the model’s reliability and enhancing the overall credibility of their conclusions. Only a few minor suggestions are included in this review.

**Major Comments: None**

**Minor Comments:**

**1. Model Limitation**

- **Normalized hill equation**

Consider briefly elaborating on the advantages of using the normalized Hill equation — such as its ability to represent saturation effects and cooperative binding — especially in the absence of precise kinetic data.

- **Parameter Sensitivity using Latin Hypercube Sampling (LHS)**

To further strengthen the parameter sensitivity analysis, you might consider incorporating Latin Hypercube Sampling (LHS). This approach is well-suited for exploring high-dimensional parameter spaces and could offer additional insight into how variations in the assumed Hill-type parameters influence model outputs, particularly given the lack of quantitative data for individual reactions.

**2. Physiological Implications**

- **References**

In both the up-regulation and knockdown analysis paragraphs, please add more citations from previously published literature on the role of CaMKII, eNOS, and GSK3β in cardiomyocytes.

**3. Possible Future Direction**

- **Personalization of Input Nodes**

The activation or deactivation of T, AngII, E2, and ET-1 nodes may vary based on sex-specific transcriptomic signatures observed at the single-cell level. These signatures reflect gender-differentiated gene expression patterns, which can influence the sensitivity and response of cells to these hormonal stimuli, ultimately leading to sex-dependent modulation of cellular behaviors such as hypertrophy. Incorporating these variations into the model could provide a more accurate representation of how sex influences the signaling dynamics in cardiac cells. This integration could also help identify novel sex-specific therapeutic targets, ultimately improving the precision of future treatments for cardiac hypertrophy.

**Recommendation: Yes**
